# The impact of land use and rainfall patterns on the soil loss of the hillslope

**DOI:** 10.1038/s41598-021-95819-5

**Published:** 2021-08-11

**Authors:** Xianmeng Meng, Yan Zhu, Maosheng Yin, Dengfeng Liu

**Affiliations:** 1 School of Environmental Studies, China University of Geosciences, Wuhan, 430074 China; 2 School of Environmental Science and Engineering, Southern University of Science and Technology, Shenzhen, 518055 China; 3 School of Water Resources and Hydropower, Xi’an University of Technology, Xi’an, 710048 China

**Keywords:** Environmental impact, Hydrology

## Abstract

In order to discuss the effect of rainfall patterns and land use types on soil erosion, the experiment is carried out under natural rainfall events on different kinds of runoff plots in Zhangjiachong watershed. Based on the observed data of 44 individual rainfall events including moderate, heavy and storm rainfall, the differences of erosion modulus among hedgerows plots, terrace plots, and slope plots under different rainfall patterns are analyzed. And the effects of hedgerow and terrace patterns on control of soil loss are revealed by RUSLE. Wilcoxon signed rank test is applied to analyze the significant difference of erosion modulus in different plots and the coefficient of variation is used to compare the characteristics of erosion modulus under different rainfall patterns. The results show that the soil erosion modulus of earth banked terrace has the highest value and the lowest soil erosion modulus occurs in the slope land with hedgerows. The coefficients of variation for soil erosion modulus under heavy and storm rainfall are larger than that of moderate rainfall. Hedgerow pattern can effectively control soil erosion under moderate and heavy rainfall while the effect of hedgerow is considerably weakened under storm rainfall. Earth banked terraces own the highest erosion modulus followed by slope land and stone dike terraces.

## Introduction

Soil erosion is a kind of environmental problems meaning soil washed away by running water, blown away by wind or removed by human activities^1^. It has brought a lot of negative effects on soil conditions such as the decline of soil quality and the decrease of crop production^2,3^. Water erosion caused by excessive rainfall has been acknowledged as one of the most common types of soil erosion significantly affecting approximately 751 Mha of land in the world^4^. In China, about 56% of lands are reported suffering from water erosion^5^. Because of the limited land resources and large amount of population, sloping farmland is used to alleviate the pressure of cropping. Meanwhile, unreasonable management measures of agriculture accelerates soil erosion^6^. Therefore, it is very essential to carry out the research on the mechanism of soil erosion and analyze the characteristics of soil erosion under different conditions.

Soil erosion is a complicated physical process influenced by natural and anthropogenic factors. Rainfall characteristics and land use management are two main factors affecting the magnitude of runoff and erosion^7–9^. As rainfall is the major driver of soil erosion which has direct impact on separation of soil particles, decomposition of soil aggregates and migration of eroded sediment, the amount of soil erosion caused by erosive rainfall accounts for most of the total erosion. Moderate rainfall with the characteristics of low intensity and long duration often causes interflow, while surface runoff is the main mode of runoff under storm rainfall event, which may damage the structure of soil and cause serious soil loss. Rainfall patterns are crucial to the shape of the runoff sediment hydrographs^10^. The time distribution of storm and rainfall pattern with high intensity, short duration and high frequency are the main reasons for runoff and sediment generation^11^. However, these rainfall patterns do not always cause runoff and transport from the place of disaggregation to other places in the landscape. The generation of soil loss depends on a volume of runoff sufficient to transport particles and this can be provided by surface sealing, the antecedent unit and soil compaction^12,13^. For cultivation of steep lands, proper management practices can effectively reduce soil erosion through intercepting rainfall, decreasing the energy of drop and increasing infiltration. Contrarily, improper land use will accelerate soil erosion.

Terraces have a significant effect on hydrological processes because the distribution and characteristics of the soil are altered by slope reduction. Besides, terraces provide flat surfaces and deep, loose soils that increase infiltration and reduce runoff^14^. And soil loss will be effectively controlled when the terraces are covered by dense grass and scrub. Vegetation cover can make a large contribution to prevent soil erosion^15^. Sediments are accumulated in front of the hedgerow belt, resulting from the slower runoff and the formation of backwater stirps above the hedgerows. Vetiver grass is often used on steep lands to reduce runoff and soil loss^16^. By doing so, initial runoff time can be delayed and peak runoff rate and erosion rate are reduced. For a small catchment, it is crucial to estimate response time of peak discharge^17,18^. In addition, the reduction of slope gradient produced by hedgerows can gradually make natural terraces on slope lands, which in turn significantly decrease runoff and soil erosion^19^.

Hilly areas of the upper reaches of the Yangtze River, especially the Three Gorges Reservoir Area, are high incidence areas of soil erosion in the Yangtze River Basin. Soil and water loss in the upper reaches of the Yangtze River Basin has a territory of 496.3 thousand km^2^ and mean annual soil erosion quantity of 2.179 billion tons^20^, respectively. These soil and water loss areas mainly distribute in the lower reaches of Jinshajiang River, Jialingjiang and Tuojiang basins, east of Sichuan Province and the Three Gorges Reservoir Region. As 96% of the Three Gorges Reservoir Area consists of mountains and hills, most of the farmlands in this area are sloping fields which show poor erosion resistance^21^. Since the construction of the Three Gorges Dam in 1990s, a lot of low-slope cultivated lands have been submerged, leading to the exploitation of more sloping lands and acceleration of soil and water erosion^22–24^.

As far as we know, most of the existing researches are based on artificial rainfall simulation experiment and studies on soil erosion under natural rainfall are lacking in most areas. As the energy produced by the rainfall simulators is low, the rainfall simulators cannot mimic natural rainfall very well in erosion simulation^25^. In addition, underlying surfaces in natural conditions with various kinds of types are more complicated than those in laboratory. Therefore, characteristics of sediment production obtained from natural conditions can provide more reliable data and reflect erosion situation more realistically.

Substantial efforts have been spent on the development of soil erosion models^26^. These soil erosion models can be classified into three groups^27^, which are empirical, conceptual (partly empirical/mixed) and physically-based. Examples for first two groups comprise the empirical USLE (Universal Soil Loss Equation) and its modifications, and few comprehensive models like ANSWERS (Areal Nonpoint Source Watershed Environment Response Simulation), CREAMS (Chemicals, Runoff and Erosion from Agricultural Management Systems), and MODANSW (Modified ANSWERS). ANSWERS and CREAMS are basically conceptual and event based models. One of the most widely applied empirical models for assessing the sheet and rill erosion is the Universal Soil Loss Equation (ULSE). The Universal Soil Loss Equation (USLE), an empirical soil erosion model, was proposed by Wischmeier and Smith^28^ for the prediction or assessment of soil erosion. Then, in order to consider the soil erosion factors more comprehensively, Renard et al.^29^ improved the traditional USLE and developed a revised Universal Soil Loss Equation (RUSLE).

Numerous researches have been conducted to investigate the controlling factor of soil loss^30–33^. However, relatively rare experimental researches have been reported under natural condition. In this study, the experiment is carried out under natural rainfall events on different kinds of runoff plots in Zhangjiachong watershed to discuss the effect of rainfall patterns and land use types on soil erosion in the Three Gorges Region in China. Detailed data were collected from systematic field experiments for a long time, which provides direct information for the research of soil loss.

## Materials and methods

### Study area

**Figure 1:**
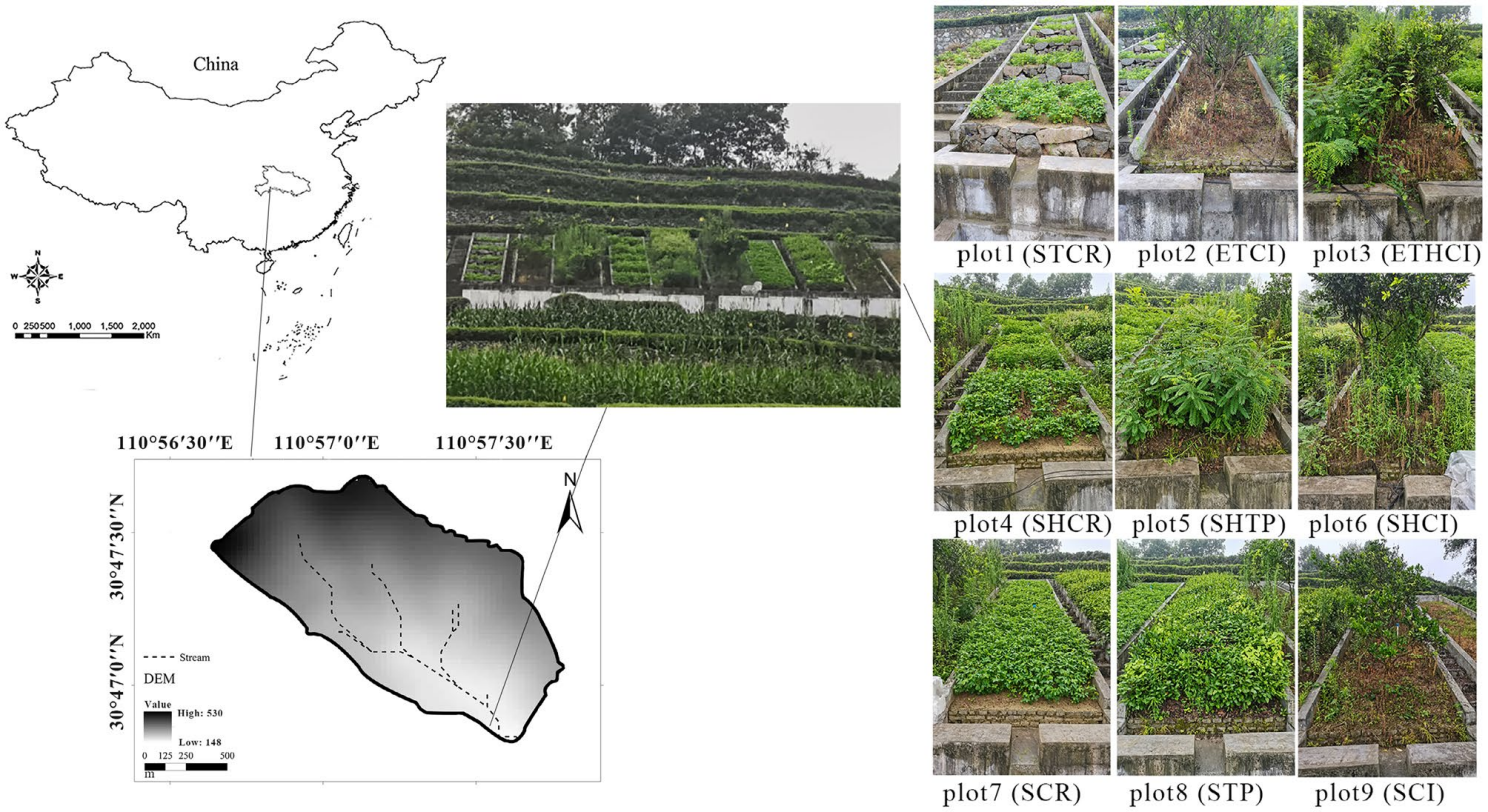
The location of Zhangjiachong watershed and runoff plots.

The study area is Zhangjiachong watershed located in the southwest of Hubei Province, China at 30°46′51′′′ N latitude and 110°57′20′′ E longitude (Fig. 1) with an area of 1.62 km^2^. This region is predominated by low-slope hilly topography with a subtropical wet monsoon climate. The average annual temperature and precipitation are 18℃ and 1439 mm, respectively^41^. And most of precipitation falls during the period from May to September. Geological properties include granite and quartz sandy soil from granite parent. The main land uses are farmland—26.7%, forest—60.6%, economic fruit forest—4.6%, grassland—2.0% and barren hills—4.9%. Soil and water loss mainly occurs in sloping lands and the areas with low vegetation coverage. Waste land and hillside with slope of over 25° especially suffer from water loss and soil erosion.

### Data collection

Data were collected from nine experimental runoff plots (Fig. 1) including stone dike terraces—crops (STCR), earth banked terraces—citrus (ETCI), earth banked terraces with hedgerows—citrus (ETHCI), slope land with hedgerows—crops (SHCR), slope land with hedgerows—tea plants (SHTP), slope land with hedgerows—citrus (SHCI), slope land—crops (SCR), slope land—tea plants (STP) and slope land—citrus (SCI). Anti-seepage walls were built with concrete along the borders of these plots to prevent water outside. Concrete tanks were constructed in the terminal of each plot. Then, the runoff and sediment can be conveniently collected from plot during rainfall events. The area of each runoff plot was 20 m^2^with slope of 25°. Five ridges were set in the earth-banked terraces and stone dike terraces.

The precipitation was continuously monitored by means of a pluviograph. As runoff and soil loss occurred almost simultaneously, runoff had to be divided into two categories: muddy water runoff and clean water runoff. The former was directly measured at the end of each rainfall event from concrete tanks. Then the latter was calculated using the former subtracted by the volume of sediment and corrected by coefficient. 44 individual rainfall events were selected including 5 moderate rainfall events, 21 heavy rainfall events, and 18 storm rainfall events according to the grade of maximum 24-h rainfall in the Grade of Precipitation (GB/T 28592-2012), which is the national standard of China (Table 1).

**Table 1:** Grade of precipitation.

Grade of precipitation	Maximum 24-h rainfall (mm)
Moderate rainfall	10–24.9
Heavy rainfall	25.0–49.9
Storm rainfall	50–99.9

## Methods

### Statistical analysis

Two kinds of mathematical statistical parameters are used to analyze the soil loss of different land use types under different rainfall patterns. One parameter is the mean value of soil erosion modulus expressed as:

1 $$\overline{{M_{ij} }} = \frac{1}{n}\sum\limits_{k = 1}^{n} {M_{ijk} }$$

where $i$ is the number of runoff plots, $j$ indicates the land use type, $n$ is the total number of some type of rainfall events and $M_{ijk}$ is the soil erosion modulus in *i*th runoff plot under *j*th land use type.

The other parameter is the coefficient of variation expressed as:

2 $$C_{v} = \frac{{\sqrt {\sum\limits_{k = 1}^{n} {(M_{ijk} - \overline{{M_{ij} }} )^{2} {\raise0.7ex\hbox{${}$} \!\mathord{\left/ {\vphantom {{} n}}\right.\kern-\nulldelimiterspace} \!\lower0.7ex\hbox{$n$}}} } }}{{\overline{{M_{ij} }} }}$$

As the data of soil erosion modulus under different types of rainfall events are not normally distributed, the Wilcoxon signed rank test^34^ is used to test whether the values of erosion modulus from different runoff plots differ from each other and make a comparison between the values of erosion modulus from different runoff plots under different rainfall patterns through SPSS 25.0 for Windows.

### RUSLE equation

As the structure of RUSLE is comparatively simple, it has become most widely used approach to assess the effects of land use on control of soil loss^35,36^. In addition, this method has been applied in the Three Gorge Area of China and verified for its effectiveness by Shi et al.^37^. Therefore, RUSLE is chosen to analyze the reason causing the soil loss of different land use types under different rainfall patterns. The RUSLE can be written as

3 $$A = R \times K \times L \times S \times C \times P$$

where $A$ (t/ha) is the soil loss for a given period; $R$ (MJ mm/ha h) is the rainfall-runoff erosivity factor; $K$ (t h/MJ mm) is the soil erodibility factor; $L$ and $S$ represent the slope length factor and the slope steepness factor respectively reflecting the topographical condition; $C$ and $P$ represent the vegetation cover factor and the conservation support-practices factor respectively, with their values dimensionless.

In this study, soil loss of different land use types are compared under certain rainfall patterns, so, it is assumed that $R$, $K$, $L$ and $S$ values in Eq. (1) are the same. The RULSE equation can be simplified as

4 $$A = t \times C \times P$$

where $t$ is a fixed combination of $R$, $K$, $L$ and $S$.

Vegetation cover is a sensitive factor affecting soil erosion as vegetation plays a very important role in controlling the process of runoff generation. The relationships between soil loss and canopy/surface cover in the Three Gorges Area of the Yangtze River have been obtained from 30 runoff-erosion plots under natural and simulated rainfall events^38,39^. And the cover and management factor (*C*) in RUSLE equation can be expressed as follows:

5 $$C = \left\{ {\begin{array}{ll} {1,} & {c = 0} \\ {0.6508 - 0.343\log c,} & {0 < c < 78.3\% } \\ {0,} & {c \ge 78.3\% } \\ \end{array} } \right.$$

where *c* represents canopy/surface-cover expressed in %.

Considering the temporal variation characteristics of rainfall and the change of vegetation cover, the value of *C* for any cropping sequences can be calculated using the monthly measured average *c* and weighted by the distribution of the rainfall runoff erosivity^37^. The values of *C* for common crop rotations with seasonal cropping sequences in one year are based on the results from Yang and Shi^38^ and Cai^39^.

The definition of the conservation support-practice factor (*P*) is the ratio of soil loss with a given surface condition to the corresponding soil loss with up-and-down slope tillage^29^. The values of *P* are obtained from experimental data^38–40^.

## Results

### Rainfall characteristics

**Figure 2:**
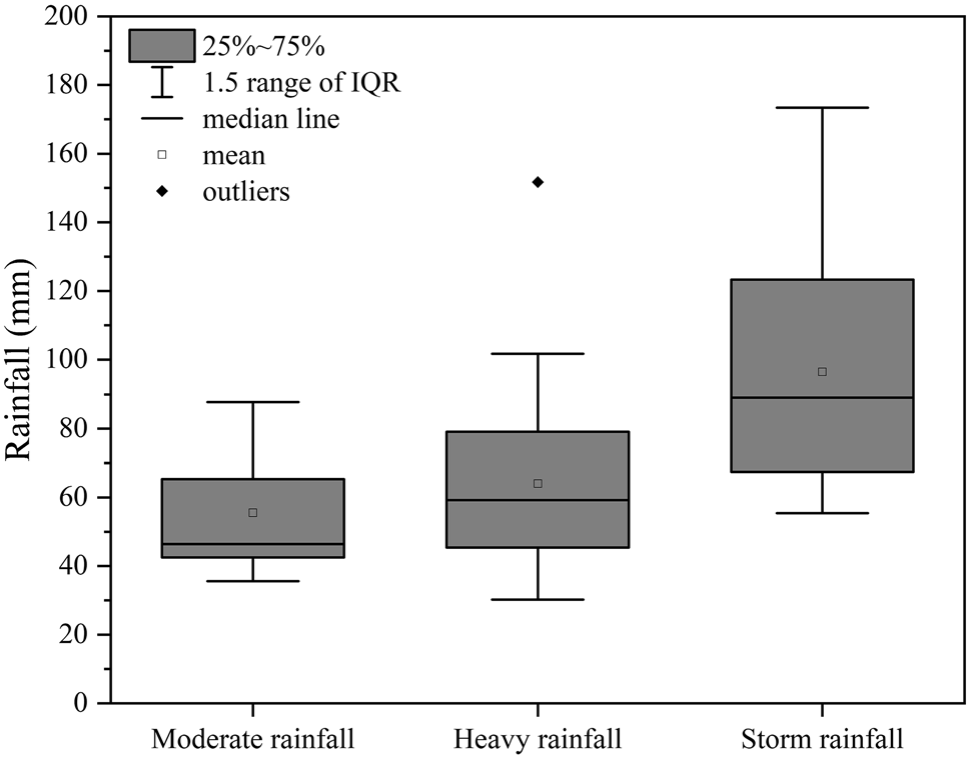
Box plot for moderate rainfall, heavy rainfall and storm rainfall respectively.

Table 2 shows statistics of 44 erosive rainfall events occurred in 2004–2006 and 2008–2014 including the amount of rainfall, the duration of rainfall and maximum 24-h rainfall. The box-plot graphics for these rainfall events are exhibited in Fig. 2. It can be seen that the characteristics of these rainfall events differ considerably: storm rainfall events have the highest mean values of the amount of rainfall and maximum 24-h rainfall followed by heavy rainfall events and moderate rainfall events, whereas the duration of rainfall exhibits the opposite trend.

**Table 2:** Statistical features of different rainfall events.

Rainfall events	Eigenvalues	Mean	Standard deviation	Variation coefficient	Frequency (times)
Moderate rainfall	P/mm	55.3	19.0	0.34	5
	D/h	240.37	172.99	0.72	
	P_24_/mm	17.1	2.8	0.17	
Heavy rainfall	P/mm	62.4	28.2	0.45	21
	D/h	132.81	170.59	1.29	
	P_24_/mm	36.63	7.0	0.19	
Storm rainfall	P/mm	96.5	34.4	0.36	18
	D/h	81.73	83.22	1.02	
	P_24_/mm	62.6	9.6	0.15	

P, D, P_24_ represents rainfall, rainfall duration and the maximum 24-h rainfall respectively.

### Runoff and soil loss of different land use types

**Figure 3:**
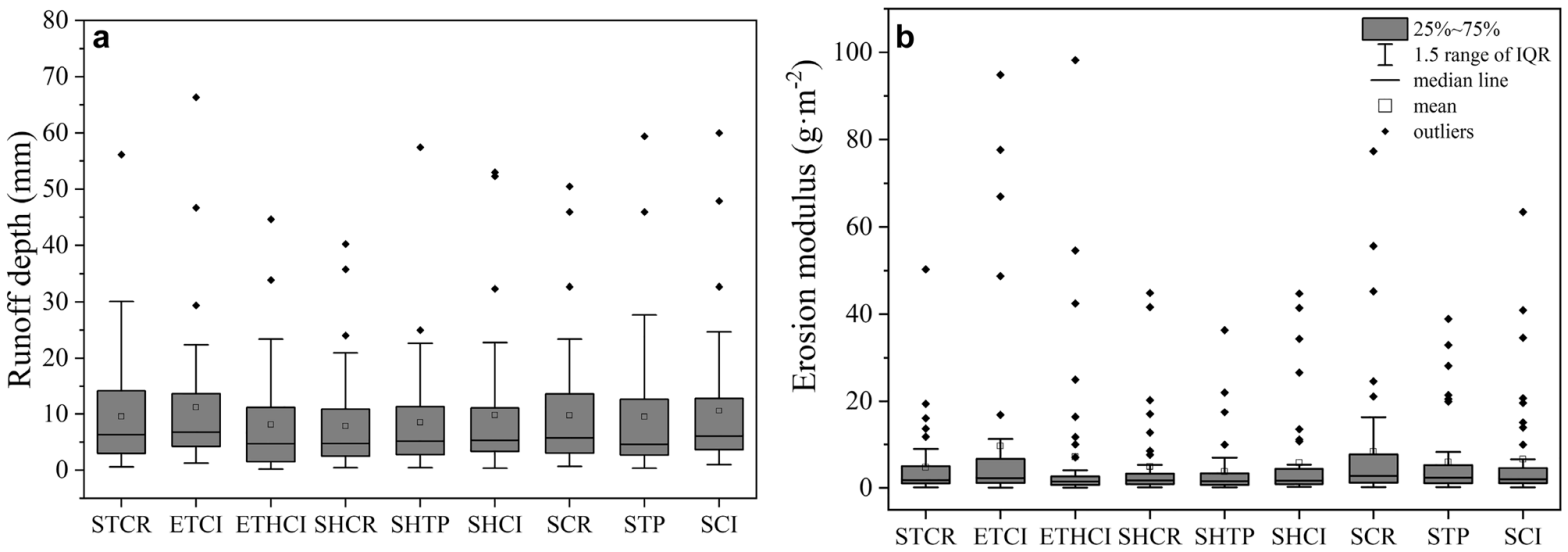
The values of runoff depth (**a**) and erosion modulus (**b**) from different runoff plots.

The box-plot graphics for runoff and soil loss of different runoff plots are exhibited in Fig. 3. The number of extreme runoff depth from each runoff plot is less than 3. The ranked order of mean values of runoff depth from these runoff plots is as follows: ETCI > SCI > SHCI > SCR > STCR > STP > SHTP > ETHCI > SHCR. The results show that hedgerow has an obvious effect on reduction of runoff.

Compared with the number of extreme runoff depth, extreme soil loss events become more frequent. The ranked order of mean values of erosion modulus from these runoff plots is as follows: ETCI > SCR > ETHCI > SCI > STP > SHCI > SHCR > STCR > SHTP. The pairs STCR vs SCR, SHCR vs SCR, SHTP vs STP, ETHCI vs ETCI indicate significant differences (p < 0.05) in soil loss characteristics (Table 3). The results show that stone dike terrace and hedgerow have a significant effect in reducing soil loss. STCR has a mean value of erosion modulus of 4.73 g/m^2^, which is 43.3% lower than SCR. SHCR has a mean value of erosion modulus of 4.92 g/m^2^, a decrease of 41.0% compared with SCR. SHTP generates a mean value of erosion modulus of 3.73 g/m^2^, which is 37.3% lower than STP. The mean value of erosion modulus of ETHCI decreases by 25.8%, in comparison with ETCI. However, the construction of earth banked terrace exacerbates soil erosion. For instance, the mean values of erosion modulus of ETCI and ETHCI increase by 45.1% and 24.0% respectively compared with SCI and SHCI.

**Table 3:** The results of the significant differences between erosion modulus of different plots obtained by the Wilcoxon signed rank test (p < 0.05, n = 44).

Wilcoxon signed—rank test			
	Pair	Reduction ratio	Check value = 0.05
			2 − Tail sig
Erosion modulus	SHCR vs SCR	41.0%	0.001*
	SHTP vs STP	37.3%	0.009*
	SHCI vs SCI		0.126
	ETHCI vs ETCI	25.8%	0.003*
	STCR vs SCR	43.3%	0.002*
	ETCI vs SCI		0.082
	ETHCI vs SHCI		0.06

*Represent p < 0.05.

The RUSLE is used to understand the soil loss characteristics of different runoff plots mentioned above. In Eq. (5), the cover and management factor is negative to canopy/surface cover, so the soil loss given in Eq. (4) is negative to canopy/surface cover. The existence of Hedgerow enhances canopy/surface cover and therefore reduces the soil loss of SHCR, SHTP and SHCI. In addition, the canopy of citrus is higher than that of crops making the erosion modulus of SCI smaller than that of SCR. The reason is that the values of *C* for rape/corn (SCR) and oranges (SCI) are 0.32 and 0.13 respectively and both of *P* values for SCR and SCI equal 1.00. Compared with slope land, stone dike terraces can effectively reduce soil loss. It is because the value of *P* for slope land is 1.00 and 0.50 for level terrace. And the soil loss of STCR and SCR is 0.16*t* and 0.32*t* respectively. This means that the soil loss of terraces could be reduced by 50% compared with slope land, which is corresponding to the conclusion obtained in the above paragraph. However, as far as earth banked terraces is concerned, the soil loss characteristics cannot be explained by RULSE.

### Runoff and soil loss of different land use types under different rainfall patterns

**Figure 4:**
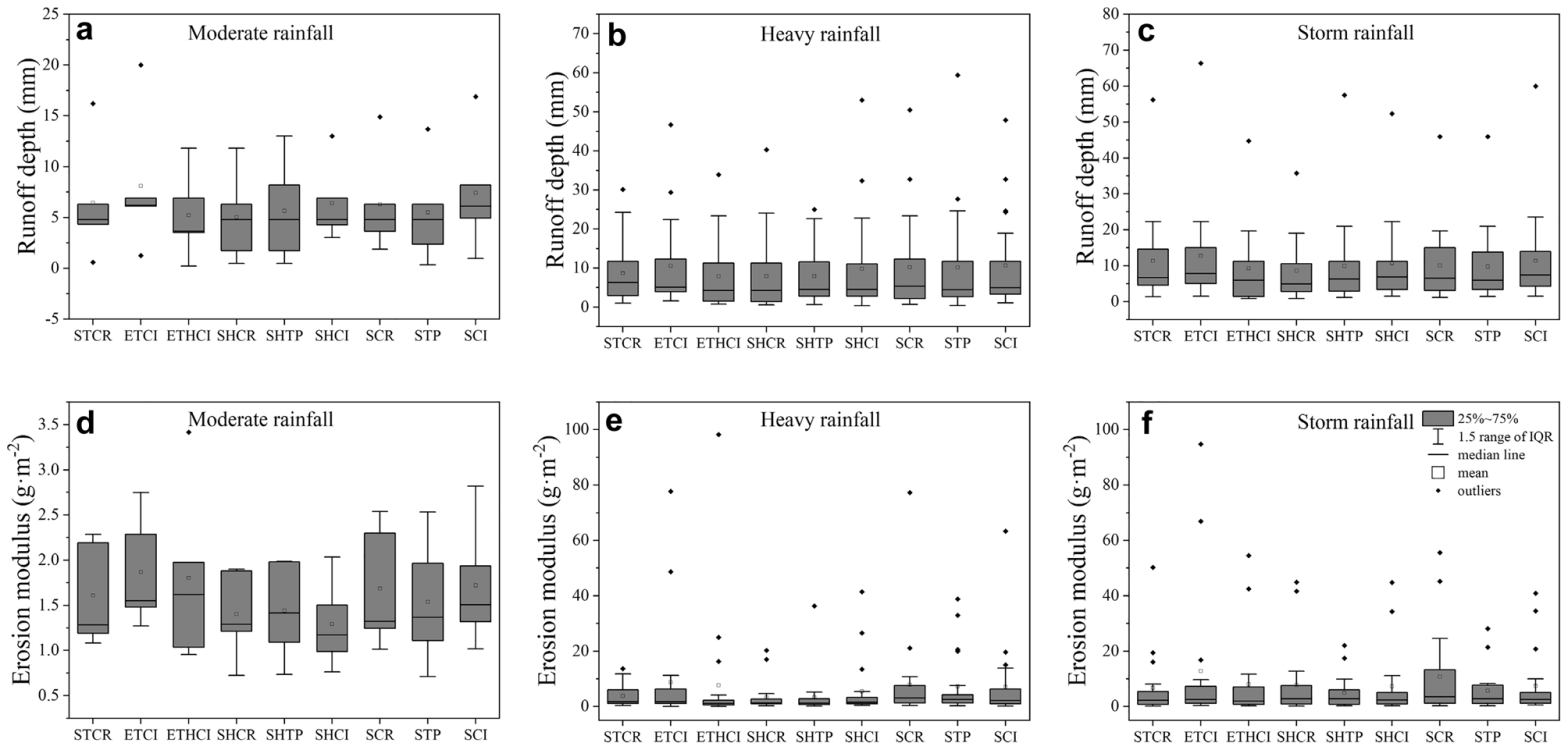
(**a**) The values of runoff depth (**a**–**c**) and erosion modulus (**d**–**f**) from different land use types under different rainfall patterns. (**a,d**) Moderate rainfall. (**b,e**) Heavy rainfall. (**c,f**) Storm rainfall.

The box-plot graphics for runoff and soil loss from different land use types under different rainfall patterns are shown in Fig. 4. The results show that the mean values of runoff depth from SHCR, SHCI, SCR, STP and SCI under heavy rainfall (Fig. 4b) are higher than that under storm rainfall (Fig. 4c). And all these runoff plots have the same ranked order of mean values of erosion modulus: storm rainfall > heavy rainfall > moderate rainfall except STP with the highest erosion modulus under heavy rainfall. Compared with moderate rainfall, heavy rainfall and storm rainfall are more likely to cause serious soil loss.

For moderate rainfall, the effect of hedgerows on soil loss control is not obvious (Fig. 4d), whereas hedgerows can effectively reduce soil loss under heavy rainfall (Fig. 4e). The results indicate significant differences in erosion modulus among the pairs SHCR vs SCR, SHTP vs STP (p < 0.05) under heavy rainfall (Table 4). In slope land with crops and tea plant, hedgerows reduce soil erosion by 58.3% and 54.0% respectively when compared to no hedgerows. When storm rainfall occurs, the effect of hedgerows on soil loss control is significantly weakened compared with heavy rainfall (Fig. 4e,f). For instance, hedgerows reduce soil erosion by 2.6% compared to no hedgerows in slope land with citrus.

**Table 4:** The results of the significant differences between erosion modulus of different plots under different rainfall patterns obtained by the Wilcoxon signed rank test (p < 0.05, n = 5, 21, 18).

Wilcoxon signed—rank test				
	Rainfall types	Pair	Reduction ratio	Check value = 0.05
				2 − Tail sig
Erosion modulus	Moderate rainfall	SHCR vs SCR	16.7%	0.043*
		SHTP vs STP		0.686
		SHCI vs SCI		0.225
		ETHCI vs ETCI		0.686
		STCR vs SCR		0.225
		ETCI vs SCI		0.5
		ETHCI vs SHCI		0.893
	Heavy rainfall	SHCR vs SCR	58.3%	0.021*
		SHTP vs STP	54.0%	0.004*
		SHCI vs SCI		0.274
		ETHCI vs ETCI	12.1%	0.021*
		STCR vs SCR	52.1%	0.03*
		ETCI vs SCI		0.205
		ETHCI vs SHCI		0.03*
	Storm rainfall	SHCR vs SCR	27.2%	0.031*
		SHTP vs STP		0.5
		SHCI vs SCI		0.372
		ETHCI vs ETCI		0.053
		STCR vs SCR		0.053
		ETCI vs SCI		0.248
		ETHCI vs SHCI		0.446

*Represent p < 0.05.

The effect of stone dike terraces on soil loss control is similar to that of hedgerows. Under moderate rainfall, the mean values of erosion modulus from STCR and SCR are nearly the same indicating slight effect on soil loss control. The mean values of erosion modulus from STCR are 52.1% (p < 0.05) lower than that of SCR under heavy rainfall showing a good performance in soil loss control. And the effect of stone dike terraces on soil loss control under storm rainfall is not significantly improved compared with heavy rainfall. In addition, earth banked terraces exacerbate soil erosion no matter which kind of rainfall pattern occurs.

The above results show that the vegetation cover factor (C) and the conservation support-practice factor (P) expressed in RUSLE have an important impact on soil loss control. Statistical features of erosion modulus for runoff plots under different rainfall patterns are shown in Table 5. The results indicate an overall trend in the mean values and standard variations of erosion modulus for all these runoff plots: storm rainfall > heavy rainfall > moderate rainfall except in the case of STP. And the coefficients of variation for erosion modulus exhibit the order of heavy rainfall > storm rainfall > moderate rainfall except STCR and SHCR. Runoff plots for Earth banked terraces have the largest mean values, standard deviations and coefficients of variation for erosion modulus followed by runoff plots of slope land and stone dike terraces.

**Table 5:** Statistical features of erosion modulus for runoff plots under different rainfall patterns.

Conservation type	Moderate rainfall		Heavy rainfall		Storm rainfall	
	Standard deviation/g m^−2^	Coefficient of variation	Standard deviation/g m^-2^	Coefficient of variation	Standard deviation/g m^-2^	Coefficient of variation
STCR	0.52	0.32	3.84	1.02	12.72	1.90
ETCI	0.56	0.30	18.44	2.11	26.81	2.10
ETHCI	0.89	0.50	21.09	2.75	16.01	2.01
SHCR	0.44	0.32	5.11	1.56	14.09	1.81
SHTP	0.49	0.34	7.50	2.25	6.34	1.31
SHCI	0.44	0.34	9.92	1.79	12.94	1.79
SCR	0.61	0.36	16.22	2.06	16.55	1.55
STP	0.64	0.42	10.81	1.49	7.86	1.39
SCI	0.63	0.36	13.64	1.92	12.70	1.71

## Discussion

### Effect of different land use types on soil loss

From these above results, it could be seen that different land use types cause different soil loss characteristics. Generally speaking, hedgerow and stone dike terraces play a key role in soil loss control while earth banked terraces exacerbate soil erosion. This conclusion is the same as the results of Shen et al.^41^ indicating that hedgerow is the best way to control soil loss followed by stone dike terraces, and the erosion modulus of earth banked terraces is higher than that of slope land.

Hedgerow reduces the soil’s susceptibility to erosion from two aspects: vegetation cover and roots. Firstly, hedgerow creates vegetation cover for protection of soil surface, which can reduce splash erosion by rainfall interception^42,43^ and decrease overland flow^44,45^. Raya et al.^46^ proved that the most effective vegetation coverage reduced soil loss by 97% compared to bare soil. Secondly, vegetation roots stabilize the soil aggregates by physically binding soil particles together and improve soil physical properties by binding agents exuded from roots and residue^47,48^. In addition, the existence of vegetation roots enhances the ability of precipitation infiltration into the soil^49,50^. Tan^51^ reported that approximately 90% of the total rainfall was held by the litter layer and soil, which eventually was exhausted by evaporation.

For slope lands, SCR has larger erosion modulus compared with STP and SCI. It is because tea plants and citrus belong to perennial shrub, whereas crops are often annual herbaceous plants. During the process of crops planting, the structure of soil in SCR is disturbed and the vegetation is destroyed by tillage, which leads to larger erosion modulus^52,53^.

Compared with slope land, stone dike terraces can also notably reduce runoff^54^ and soil loss^55^. By analyzing the runoff and sediment regulation mechanism of the terraces, it is revealed that long slope existed in slope land is changed by terraces into several short slopes. This landform change makes the sediment generation from itself more difficult and intercepts the sediment yield from the upper area^56–59^. The sediment reduction potential of level terrace can be up to 65%^60^. Earth banked terraces increase soil loss. It may be ascribed two reasons. One the one hand, earth banked terraces cannot be constructed high enough because of the limited length of plots. Thus, the area of bare land is significantly increased and the surface flow cannot effectively slow down, which leads to more eroded soil^41^. On the other hand, many rills are observed on bare earth banked terraces constituting the primary form of erosion. And some main rills have developed into gullies running through the upper terrace and the lower terrace, which exacerbates soil erosion^61^.

### Soil loss response to different land use types under different rainfall patterns

The study area belongs to typical monsoon climate region. The characteristic parameters to identify the feature of moderate, heavy and storm rainfall are not the total amount of precipitation but the duration and intensity. Moderate rainfall events, often monsoon rains, which develop due to annual variations in temperature difference between continents and oceans, occur in spring and autumn with the feature of long duration and low intensity. Heavy rainfall and storm rainfall events, which are caused by local convective activities, usually happen in summer with short duration and high intensity. Compared with moderate rainfall events, heavy rainfall and storm rainfall events own a greater destructive power to destroy the structure of soil and cause larger erosion modulus. And the values of soil erosion modulus are more sensitive to land use types under heavy rainfall and storm rainfall events.

The effect of hedgerows on soil loss control in slope lands under heavy rainfall is better than that under moderate rainfall and storm rainfall (Fig. 4d–f). It is because moderate rainfall events with low runoff and rainfall erosivity often cause limited sediment yield. When storm rainfall events occur, the soil moisture of hedgerow slope is usually saturated by antecedent rainfall leading to the reduced erosion resistance. In addition, the hedgerow belt may be destroyed by extreme rainfall with high intensity^62^.

For the earth banked terraces, the effect of hedgerow on soil loss control is becoming more and more significant with the increasing of rainfall intensity (Fig. 4d–f). This result is similar to previous studies on hedgerow effects on soil sediment transport obtained by Liu et al^63^, who observed soil erosion increased obviously if no protective measures existed on dikes, and could even lead to soil collapse during storm rainfall events. In addition, the coefficients of variation for ETCI and ETHCI are significantly higher than that for other runoff plots indicating that the erosion modulus of earth banked terraces is more sensitive to the initial condition of soil or other stochastic factors.

## Conclusions

In this study, the experiment is carried out under natural rainfall events on different kinds of runoff plots in Zhangjiachong watershed to discuss the effect of rainfall patterns and land use types on soil erosion in the Three Gorges Region in China. The differences of erosion modulus among hedgerows plots, terrace plots, and slope plots under different rainfall patterns are analyzed according to individual rainfall events including moderate, heavy and storm rainfall. And the effects of hedgerows and terrace patterns on control of soil loss are revealed by the revised Universal Soil Loss Equation (RUSLE).

The results show that the ranked order of mean values of erosion modulus from these runoff plots is as follows: ETCI > SCR > ETHCI > SCI > STP > SHCI > SHCR > STCR > SHTP. The pairs STCR vs SCR, SHCR vs SCR, SHTP vs STP, ETHCI vs ETCI compared by the Wilcoxon signed rank test indicate significant differences (p < 0.05) in soil loss characteristics. All these runoff plots have the same ranked order of mean values of erosion modulus: storm rainfall > heavy rainfall > moderate rainfall except STP with the highest erosion modulus under heavy rainfall.

Different land use types have a significant effect on soil erosion under different rainfall patterns. The soil erosion modulus of earth banked terrace has the highest value and the lowest soil erosion modulus occurs in the slope land with hedgerows. The coefficients of variation for soil erosion modulus under heavy and storm rainfall are larger than that of moderate rainfall. Hedgerow pattern can effectively control soil erosion under moderate and heavy rainfall while the effect of hedgerow is considerably weakened under storm rainfall. The erosion modulus of slope land is higher than that of stone dike terraces and lower than that of earth banked terraces.

This study provides useful information for policymakers and land managers involved in the promotion of large-scale implementation of suitable similar practices. Both stone dike terraces and hedgerows can effectively reduce soil loss, however, for the regions with heavy and moderate rain, the latter is preferred based on the economic consideration.

## Data Availability

The datasets generated during and/or analyzed during the current study are available from the corresponding author on reasonable request.
